# Prevention of Renal Diseases

**Published:** 1906-01

**Authors:** Wm. T. Jones

**Affiliations:** Atlanta, Ga.


### PREVENTION OF RENAL DISEASES.*

BY WM. T. JONES, M.D.

ATLANTA, GA.

Osler in his Practice on Medicine (1903 ed.) treats the diseases of the kidneys under fourteen heads, or names, with numerous subheads in a few cases, and as I know of no better order in which to discuss the "Prevention of Renal Diseases" in a systematic way I shall take them up as they occur in his book.

1. With our present knowledge of medicine little or nothing can be done to prevent *malformations* of the kidneys, but a renewed warning can be suggested to surgeons that in some cases there is only one kidney, which even if diseased had better be *left*, floating on what not whither it will.

2. As a very large per cent. of *movable kidneys* occur in women and are caused by tight lacing and the relaxation of the abdominal walls which follows repeated pregnancies, preventive meas-

*Read before the Fulton County Medical Society, December 7th, 1905.

ures are in almost as hopeless a condition here as in malformations of the kidneys. Probably by electing a president of the United States with views a little different on race suicide we might be able to improve matters somewhat. Trauma and the lifting of heavy weights are also occasional causes for movable kidneys, and here I need only to suggest the cause rather as an aid in diagnosis.

3. The *circulatory disturbances,* causing active and passive congestion of the kidneys, are the result largely in the former condition of continued exposure to cold where the skin becomes chilled, and also by irritants, such as turpentine, cubebs, cantharides and copaiba. While passive congestion is a result of chronic diseases of the heart or lungs, or may be caused by pressure upon the renal veins by tumors, the pregnant uterus, or ascitic fluid.

4. Under the *Anomalies of the Urinary Secretion* there are fourteen subheads ranging from *anuria* caused by intense congestion, blocking of the two ureters by fragments of stones, fevers, acute poisoning by phosphorus, lead, turpentine; in collapse after severe injuries or after operation, and hysterical anuria, all the way through haematuria, haemoglobinuria where rest is essential, albuminuria caused by muscular or mental overwork, cold baths, dyspepsia and on including pyuria, chyluria (non-parasitic), lithuria, oxaluria, cystimuria, phosphaturia, indecanuria, melanuria, pneumaturia and other substances, such as fat, volatile fatty acids, diacetic acid and B-opy-butyric acid.

5. *Uremia* being caused, for whatever reason, by a retention in the blood of excrementitious material—body-poisons—which should be thrown off by the kidneys, can be prevented by keeping the kidneys in their normal condition and not overtaxing them with too much or too stimulating food.

6 and 7. *Acute Bright's disease* being in many instances the precursor of the chronic form can be spoken of in the same sentence with the latter and treated at the same time. Acute Bright's is caused most commonly by exposure to cold and wet and very often follows exposure after a drinking bout. Other causes are the poisons of specific fevers, particularly scarlet fever, less commonly typhoid fever, measles, diphtheria, smallpox, chicken-pox, malaria, cholera, yellow fever, meningitis, irritating toxic agents, such as carbolic acid, chlorate of potash, cantharides, turpentine, pregnancy, extensive lesions of the skin, as in burns, in chronic skin diseases, and also after trauma and operations on the kidney. Osler says further that malaria causes some cases of acute as well as chronic forms of Bright's, and that while alcohol probably never excites an *acute* nephritis, it probably plays an important part particularly in conjunction with other factors in the chronic form. Of men who have worked hard, eaten freely and taken alcohol to excess, he says: "After forty, in men of this class, nothing is more salutary than to experience the shock brought by the knowledge of the presence of albumin and tube-casts in the urine." Syphilis and tuberculosis are also given as causes for chronic Bright's, but among the better classes in this country he considers that overeating causes more cases even than alcohol. An article that appeared in the Journal A. M. A. Nov. 4, 1905, entitled: "Recent Advances in the Physiology of Human Nutrition," by Frank Billings, of Chicago, is a strong support of this view.

